# Malaria elimination in Botswana, 2012–2014: achievements and challenges

**DOI:** 10.1186/s13071-016-1382-z

**Published:** 2016-02-24

**Authors:** Simon Chihanga, Ubydul Haque, Emmanuel Chanda, Tjantilili Mosweunyane, Kense Moakofhi, Haruna Baba Jibril, Mpho Motlaleng, Wenyi Zhang, Gregory E. Glass

**Affiliations:** National Malaria Programme, Ministry of Health, Gaborone, Botswana; Emerging Pathogens Institute, University of Florida, Gainesville, Florida USA; Department of Geography, University of Florida, Gainesville, Florida USA; Vector Control Specialist/Consultant, 11 Granite Street, Plot 33421/917 Kamwa South, Lusaka, Zambia; World Health Organization, Gaborone, Botswana; Institute of Disease Control and Prevention, Academy of Military Medical Science, Beijing, People’s Republic of China

## Abstract

**Background:**

Botswana significantly reduced its malaria burden between 2000 and 2012. Incidence dropped from 0.99 to 0.01 % and deaths attributed to malaria declined from 12 to 3. The country initiated elimination strategies in October 2012. We examine the progress and challenges during implementation and identify future needs for a successful program in Botswana.

**Methods:**

A national, rapid notification and response strategy was developed. Cases detected through the routine passive surveillance system at health facilities were intended to initiate screening of contacts around a positive case during follow up. Positive cases were reported to district health management teams to activate district rapid response teams (DRRT). The health facility and the DRRT were to investigate the cases, and screen household members within 100 m of case households within 48 h of notification using rapid diagnostic tests (RDT) and microscopy. Positive malaria cases detected in health facilities were used for spatial analysis.

**Results:**

There were 1808 malaria cases recorded in Botswana during 26 months from October, 2012 to December, 2014. Males were more frequently infected (59 %) than females. Most cases (60 %) were reported from Okavango district which experienced an outbreak in 2013 and 2014. Among the factors creating challenges for malaria eradication, only 1148 cases (63.5 %) were captured by the required standardized notification forms. In total, 1080 notified cases were diagnosed by RDT. Of the positive malaria cases, only 227 (12.6 %) were monitored at the household level. One hundred (8.7 %) cases were associated with national or transnational movement of patients. Local movements of infected individuals within Botswana accounted for 31 cases while 69 (6.01 %) cases were imported from other countries. Screening individuals in and around index households identified 37 additional, asymptomatic infections. Oscillating, sporadic and new malaria hot-spots were detected in Botswana during the study period.

**Conclusion:**

Botswana’s experience shows some of the practical challenges of elimination efforts. Among them are the substantial movements of human infections within and among countries, and the persistence of asymptomatic reservoir infections. Programmatically, challenges include improving the speed of communicating and improving the thoroughness when responding to newly identified cases. The country needs further sustainable interventions to target infections if it is to successfully achieve its elimination goal.

## Background

Recent reductions in the morbidity and mortality associated with malaria (especially *Plasmodium falciparum*) infections have encouraged many countries to consider eliminating the disease [1]. The current strategy focuses in regions where malaria is seasonally transmitted and relatively uncommon -- described as reducing disease ‘around the edges’ [2]. Detailed analyses of elimination strategies incorporating heterogeneity in transmission (in both space and time) identify changing rates of transmission, semi-immune/asymptomatic carriers and reintroduction of the parasites by either vectors or humans as substantial challenges to ongoing efforts [3, 4]. Once the testing positivity rate falls below 5 % and the annual incidence is less than five per 1000 the World Health Organization (WHO) recommends information and surveillance systems be redesigned from aggregated reporting to case-based surveillance for elimination [5]. The area of interventions should narrow to transmission foci, and ultimately to individual malaria cases. Therefore, efficient information and reporting systems become essential, making high-quality surveillance of populations in the operational zones critical. However, few recent national-level experiences examine the extent of these challenges including Botswana; where these elimination strategies have only been recently implemented.

**Fig. 1:**
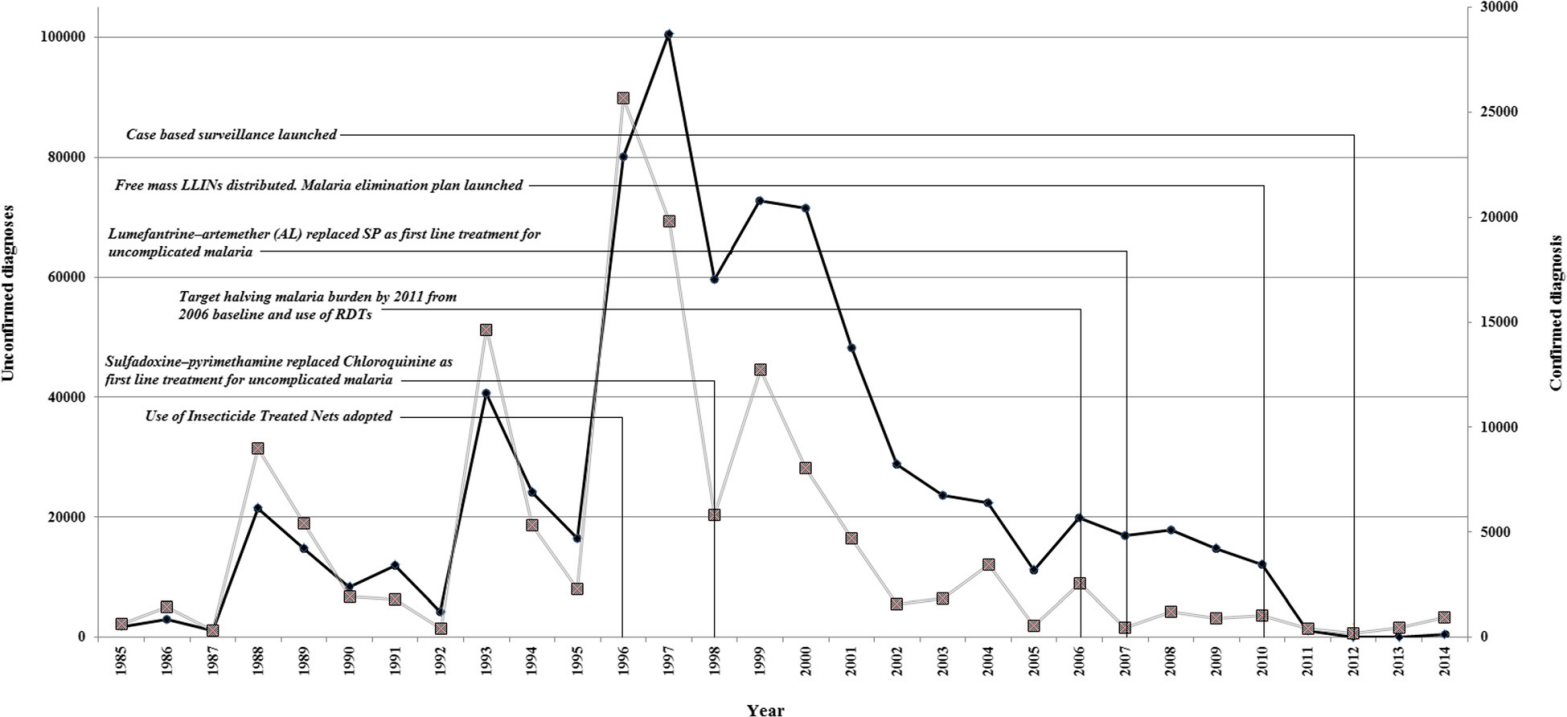
The policies and attributed changes in malaria case numbers in Botswana, 1985–2014 (Source: NMP, Botswana) [1996: Use of Insecticide Treated Nets (ITNs) adopted; 1998: Sulfadoxine–pyrimethamine (SP) replaced Chloroquinine (CQ) as first line treatment for uncomplicated malaria; 2006: Target halving malaria burden by 2011 from 2006 baseline and use of RDTs; 2007: Lumefantrine–artemether (AL) replaced SP as first line treatment for uncomplicated malaria; 2010: Free mass LLINs distributed. Malaria elimination plan launched; 2012: Case based surveillance launched]

In 1997, malaria reached a peak of 102,000 cases (62 cases/1000 population) following an outbreak in Okavango District of Botswana (Fig. 1) [6, 7]. The subsequent decline in cases could be attributed to changes in policy begun in 1996 (Fig. 1). With sustained political and financial support from the national government, Botswana has significantly reduced its malaria burden since 2000 [8]. The national malaria program (NMP) reduced cases from 17,886 (43 cases/1000 population) to 311 (0.02/1000 population) (98 %), and malaria-related deaths from 12 to 3 between 2008 and 2012 [8, 9]. Malaria transmission in Botswana is relatively very low compared to other African Countries. Nigeria for example reported the highest number of infections globally in 2013, with 12.8 million infections, followed by the Democratic Republic of Congo (11.3 million cases) and Zambia (5.5 million cases) [10].

Malaria indicator surveys conducted in 2007 and 2012 showed an increase in ownership of LLINs from 9.4 % (2007) to 52.9 % (2012)] and use of mosquito nets by children from 6.5 % (2007) to 50.6 % (2012)] [8, 11]. Additionally the population protected by IRS increased from less than 20 % in 2008 to 38 % in 2012 [8, 11]. The Okavango pilot project demonstrated that LLINs coverage increased from 13 % in 2007 to 94 % in 2010 and usage increased from 5.3 % in 2007 to 46 % in 2010 [8, 11]. Parasite surveys conducted in 2012 also found a parasite prevalence of less than one percent in 3900 children tested with RDTs [8, 11]. The deployed tools also included improved diagnosis with rapid diagnostic tests (RDTs) and treatment with Artemisinin based combination therapy (ACT) [9]. These efforts were coupled with community mobilization campaigns using electronic (radio and television) and print (newspaper) media. The NMP trained 1300 clinicians working in outpatient departments, emergency departments with an average two nurses per clinic [12]. Supplementary vector control interventions (larviciding) were occasionally used to reduce human vector contact during local outbreaks [9]. The country achieved rapid economic growth from one of the poorest countries in 1966 to a middle income country by the mid-1990s due to the diamond mining industry [13, 14]. The decline in malaria cases can also be from improved socio-economic conditions [15, 16].

In 2014, Algeria was classified as the only African country in a malaria elimination phase (and one of nine countries in the world) [10]. At the same time, Cape Verde was in pre-elimination and Egypt was in the phase of the prevention of re-introduction stage [10]. By 2012, malaria incidence rates in Botswana were sufficiently low (incidence <1 per 1000 population) meeting the WHO criterion for a malaria-free region [5]. To reach the criterion for malaria elimination, Botswana transitioned to case-based surveillance in 2012 and targeted elimination by 2015 - in line with the Southern Africa Development Community (SADC) [17]. The country has strengthened case management, rolling out case based surveillance at the national level, case management at the community level and vector control. But challenges remain to meet the WHO criteria for elimination in case management, community involvement and strengthening community surveillance. This study documents the experiences, identifies achievements of strengthened malaria surveillance during the period and challenges experienced during the early implementation of case based surveillance.

## Methods

### Study area

**Fig. 2:**
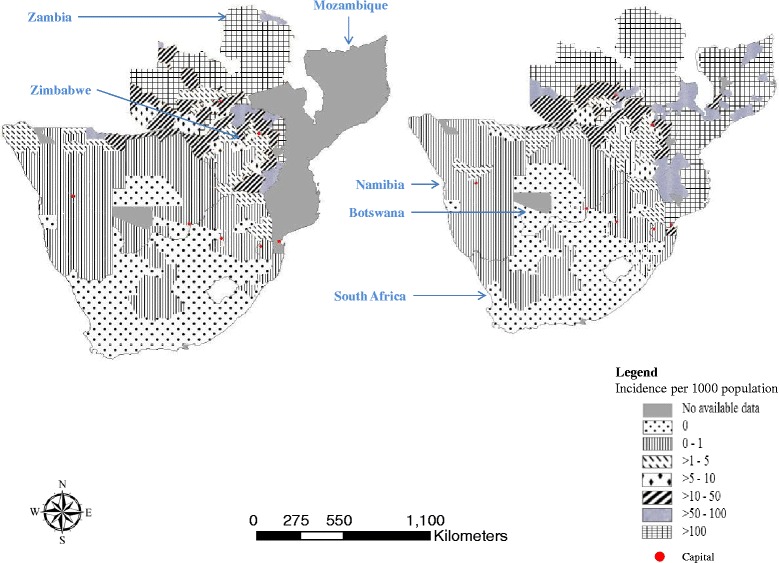
Malaria endemic countries around Botswana [59]

Botswana is a land-locked country of 581,730 km^2^ in Southern Africa bordering Namibia, South Africa, Zambia and Zimbabwe (Fig. 2) [18]. It has a mean elevation approximately 1000 m above sea level [18]. The terrain is generally flat with gentle undulations [18], and 71 % population of 2.02 million people lives along the wetter and more fertile, northern and eastern part of the country [19]. Malaria transmission in Botswana is seasonal in the Northern and Northeastern parts of the country [9]. The Southern and Western parts of the country are mainly covered by the Kalahari Desert with sparse population and have no malaria transmission [9]. Malaria foci are located in the Okavango delta and districts along the borders with Zambia, Zimbabwe and Namibia [6, 9]. *Plasmodium falciparum* is the dominant parasitic species (98 %), and *Anopheles arabiensis* is the primary vector [11].

### Data

**Fig. 3:**
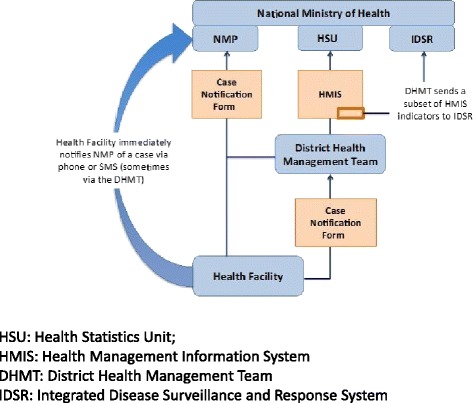
Flow of malaria data from health facility to National Malaria Program (NMP)

Malaria cases were detected using passive surveillance and were to be followed by active screening of contacts around a case during case investigation. Patients who presented with fever, chills, and flu-like illness at health facilities were tested with RDT in clinics and by microscopy in hospitals. In malaria free - southern parts of the country, health workers tested for malaria among patients with fever or patients with a history of travel to endemic areas or countries. Multiple data management systems were in place including the routine Health Management Information Systems (HMIS), Integrated Disease Surveillance and Response System (IDSR), Integrated Patient Management System (IPMS), Disease Laboratory Information Systems (DISLAB) and Logistics Management Information System (LMIS) (Fig. 3). The IPMS, a computerized data base where health workers entered information into the data base, was connected to the national level. The system was used by hospitals and it ran concurrently with HMIS. IDSR data collection was integrated into the HMIS, while IPMS was also integrated with DISLAB. HIMS ensured all patients were tallied in registers according to the diagnosis by the health worker at health facilities or as they were seen in outpatient departments at all clinics and hospitals. The data were summarized every week and reported through IDSR. The country is currently developing a District Health Information System (DHIS) to integrate all surveillance data.

Botswana introduced malaria case-based surveillance in October 2012 to strengthen reliability, completeness and timeliness of surveillance for malaria elimination. This system introduced case notification for all confirmed malaria cases and new reporting forms (see supplementary info: case notification forms, case follow up, case investigation, case screening forms, and a new algorithm for malaria diagnosis and treatment at the health facility level) to collect health, demographic, and travel history from individual malaria-positive cases in-order to capture information required for elimination.

### Routine surveillance

Botswana tested suspected cases who met the case definition in the algorithm for diagnosis and treatment (see supplementary info) at health facilities. Health workers entered suspected and tested malaria cases and deaths in a malaria register at each health facility. District Health Management Teams (DHMT) collected, compiled, analyzed and reported weekly malaria cases and deaths. In malaria free areas (a malaria free area reporting less than one confirmed malaria case per 1000 population per year) a single confirmed malaria case constituted an outbreak [5]. Health workers in malaria free areas were required to report confirmed malaria cases from all districts using standardized case-based notification forms which were sent to the DHMT and NMP.

### Reactive case detection

Confirmed malaria cases and deaths were supposed to be reported within 24 h and the completed notification forms submitted to DHMT within 48 h. These reports were intended to trigger active surveillance at and near households by health workers, using the case investigation and screening forms. This investigation identified the locality of the case - Ward/Village, Street number of household as well as the possible source of infection – stagnant water pools around household. Travel history (past 14 days) was collected on the notification form and travel history of contacts was collected during investigation. Individuals at each case household and at households up to 100 m around the index house were screened using RDTs and a case screening form was completed. Cases were classified into five categories – i. Imported case - *contracted outside Botswana*, ii. Local importation - *a malaria case traced to a different district within Botswana* iii. Introduced case - *contracted from an imported case* ie secondary case, iv. Indigenous case – *case contracted locally*, v. Induced case - *contracted locally (blood transfusion)*.

### Diagnosis and treatment

RDT (Paracheck) [20] results were used to make treatment decisions at peripheral health facilities. Microscopy was used as the gold standard for malaria diagnosis so all RDT-positive malaria cases were re-examined by microscopy by being sent to the National Reference Laboratory. Confirmed uncomplicated cases were treated with artemether-lumefantrine (AL) or quinine intravenous followed by AL if they were severe. Pregnant women during first trimester and children under 5 years were treated with quinine. The completeness of follow up for patients was recorded from records at the various facilities, with 28 days representing the expected duration for evaluation.

### Quality assurance

Laboratory technicians were trained in a WHO microscopy accreditation course to improve their skills to detect parasites. All laboratories were required to participate in proficiency testing and external quality control. Proficiency testing involved sending blinded slides from the National Reference Laboratory to the various laboratories. All staffs were required to review the slides and send their results to the national health laboratory that graded and sent the results back as part of the assessment. External quality control visits were performed by national reference staff at health facilities. Visiting staff assessed health workers who performed RDTs on site and implemented retraining to correct any problems observed in the field.

### Vector control interventions

There was one IRS cycle from October to December every year. The chemicals used were DDT (dichlorodiphenyltrichloroethane) for traditional structures (pole and dagga houses) [21] and lambda cyhalothrin ICON 10 CS [22] for modern structures (cement plastered houses). This annual routine IRS was conducted in six districts (Okavango, Ngami, Chobe, Boteti, Bobirwa and Tutume) that were hotspots of malaria transmission, with a target population of 380,000 (18.7 % of total population) in approximately 296,185 households [19]. LLINs were routinely distributed at antenatal and child welfare clinics and were distributed to achieve universal coverage in Bobirwa, Boteti, Chobe, Ngami and Tutume districts in 2010. A follow-up mass distribution campaign was conducted in Okavango district in 2011 targeting 59,421people [23]. In Okavango following the distribution a survey was conducted in collaboration with Clinton Health Access Initiative confirmed 90 % of households had at least two LLINs and 98 % had at least one LLIN [23].

### Data management, statistical analysis and mapping

Epi Info (2004) was used to analyze data collected on the notification, case investigation, case screening and follow-up forms. We performed descriptive analyses for individual cases from October, 2012 to December 2014.

Daily records from October 2012 to December, 2014 of malaria positives at health facilities were used for time series analysis (emerging hot spot analysis) in ArcGIS Pro 1.1 (ESRI, Redlands, CA). Space time pattern mining tools were used. This tool used all locations over 26 months. Based on distance and visualization, each location was assigned 25 km by 25 km spanning area. Each time step interval was 1 month in duration so the entire time period covered by the space time cube was 26 months. Of the 1089 total locations, 84 (7.71 %) contained at least one point for at least one time step interval. These 84 locations comprised 2940 space time bins of which 145 (4.93 %) had point counts greater than zero. The Emerging Hot Spot Analysis tool that relies on Getis-Ord G(i) statistic was used for space-time hot spot detection. The analysis identifies either ‘no pattern detected’ (outcome does not fall into any of the hot or cold spot patterns), ‘new’ (the most recent time step interval was statistically significant hot spot but was not previously a statistically significant hot spot), ‘oscillating’ (a statistically significant hot spot in recent time interval but has a history of also being a statistically significant cold spot during a preceding time step) and ‘sporadic’ (a location that varies as a hot spot and < 90 % of the time-step intervals have been statistically significant hot spots but never been statistically significant cold spots) hot spots. Once the space-time hot spot analysis was completed, each location (25 km by 25 km spanning area in Botswana) had an associated z-score, *p*-value, and hot spot classification (none, new, oscillating or sporadic hot spots) assigned to it.

## Ethical approval

This study was approved by the Health Research Board, Botswana.

## Results

### Malaria incidence

**Fig. 4:**
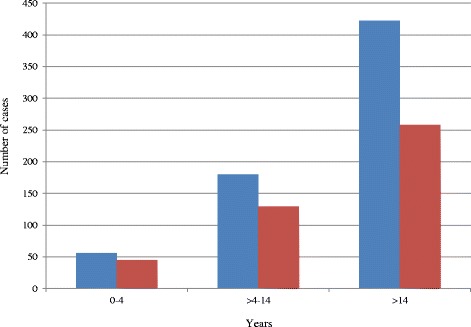
Malaria Cases by age and sex, Oct 2012–Dec 2014 (Blue bar shows male and red bar shows female)

**Fig. 5:**
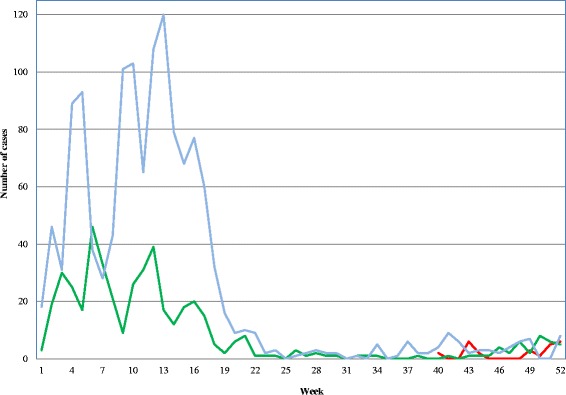
Seasonality of malaria cases in Botswana (Case-based surveillance started in October, 2012. Red color represents 2012, green 2013 and blue 2014 respectively)

Botswana reported 1808 positive malaria cases between October 2012 and December 2014. Only 1148 (63.5 %) confirmed cases were reported using the official individual notification form. Twenty seven deaths (case fatality rate = 1.5 %) were confirmed. Notified malaria cases were predominantly outpatients (75 inpatients and 1073 outpatients) and primarily adults (91 %). Only 97 (<5 years old) children were diagnosed (Fig. 4). Incidence was higher among males (59 %) (standard error: 1.71, 95 % CI: 0.55–0.61) (Fig. 4). Among female children (1–14 years) the median age was 8 years of age, and it was 32 years of age among women (>14 years). Among male children the median age was 9 years, and it was 30 years of age among men. Infection was seasonal and mostly occurred in the rainy season (December–May) with a peak from January to April (Fig. 5). Sixty nine cases were imported from outside of Botswana and 31 cases were locally imported. Between October 2012 and December 2014 there were 152 health facilities that reported at least one malaria case out of 674 health facilities. Okavango had the highest (1.91 %) incidence. Chobe, Ngami and Bobirwa district reported >0.10 % incidence.

**Fig. 6:**
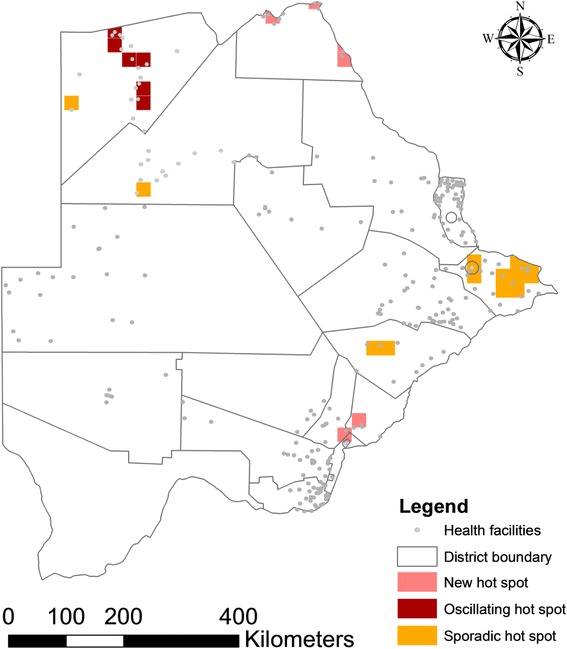
Malaria hot spots in Botswana (October 2012–December, 2014) [new hot spot: the most recent time step interval was hot and detected for the first time; oscillating hot spots: some of the time step intervals were hot, some were cold. last time step was not hot; sporadic: some of the time step intervals were hot]

The Emerging Hot Spot Analysis identified new hot spot regions on the southern and northern borders of the country (Fig. 6). Whereas oscillating hot spots were detected in Okavango district, in the north-western region of Botswana. Most sporadic hot spots were observed in eastern of Botswana.

### Malaria diagnosis and treatment

RDT was used to diagnose 1148/1808 positive malaria cases. Microscopy was used to confirm 210 cases detected by RDT and 19 (9.0 %) were reported negative. Except for 72 inpatients (admitted in hospital) who were treated with Quinine, all cases received AL. Forty nine of the 199 (24.6 %) cases which were slide positive on day zero were followed up on day 3, and 12 cases were still positive. Twenty six cases were followed up to day 14 of which 3 were still positive. Only 20 cases were followed for 28 days to confirm parasite clearance and four cases were found to be positive.

### Case investigation and screening

The surveillance guidelines required that all malaria cases be investigated at the household level [12]. Botswana NMP performed follow up surveillance for only 277/1148 (24.1 %) positive malaria cases. Screening of individuals near the 277 case households identified 37 asymptomatic (1.1 %) additional infections among 3237 individuals screened (Table 1).

**Table 1:** Cases investigated, screened and positive cases from screening at district level between October, 2012 to December, 2014

District	Cases reported through IDSR^b^	Number notified individually	Cases investigated	Total screened	Positive cases from screening
Okavango	1090	634	52	343	4
Ngami	152	74	35	421	14
Chobe	134	134	98	1026	6
Bobirwa	103	58	28	467	0
Boteti	14	44	2	20	0
Mahalapye	52	23	12	290	0
Francistown	14	6	0	0	0
Kgatleng	17	20	7	34	6
Palapye	69	39	21	484	2
Tutume	35	29	8	20	0
Gaborone	37	26	1	3	1
Serowe	26	26	6	47	0
SPTC	20	12	0	0	0
Kweneng East	20	14	6	82	4
Total^a^	1808	1148	277	3237	37

^a^Only districts reported more than five cases through IDSR has presented here (complete table: supplement section). ^b^Integrated Disease Surveillance System

### Importation

**Fig. 7:**
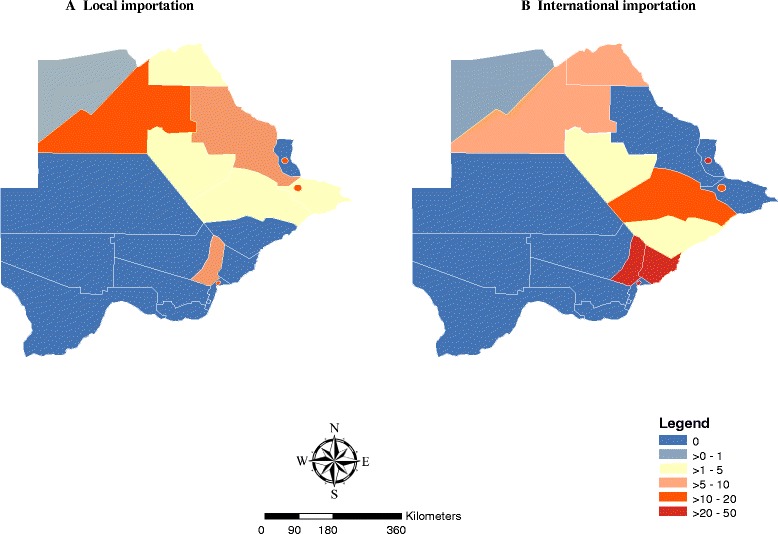
Imported malaria in Botswana (October 2012–December, 2014)

There were 961 (83.7 %) cases which were classified as locally acquired, while 69 cases were imported from other countries and 118 cases could not be classified due to inadequate information. In addition to the 69 (6 %) transnational cases, local importation accounted for 31 (2.7 %) cases. All locally imported cases reported in Ngami were from neighboring Okavango. Okavango, Ngami and Chobe reported two, eight and five imported cases from movements within Botswana (Fig. 7). Palapye reported cases from Bobirwa. Locally imported cases reported in Gaborone were from Chobe. Among the 69 transnational cases, the greatest numbers of cases were imported from Zimbabwe, Zambia, Malawi and Mozambique respectively (Table 2). Malaria free districts like Gaborone (50 %) and Kgatleng (45 %) accounted for the largest number of imported cases from outside of Botswana.

**Table 2:** Imported cases by country of origin between October, 2012 to December, 2014

Country of Origin	Number of cases (%)
DRC	2 (2.9)
Ghana	2 (2.9)
Malawi	7 (10)
Mozambique	6 (8.7)
Namibia	1 (1.5)
South Africa	2 (2.9)
South Africa	1 (1.5)
South Sudan	1 (1.5)
Zambia	19 (27)
Zimbabwe	28 (41)
Total	69 (100)

### Epidemic

During the monitoring interval several outbreaks occurred within the country and most notably in Ngami [Incidence rate ratio (IRR) - 13], Okavango (IRR - 198) and Tutume (IRR - 11) reported severe outbreaks. Approximately 60 % (Standard error: 1.77, 95 % CI: 0.57–0.63) of the country’s cases reported were from Okavango district, alone.

## Discussion

Data from the two and a quarter years of case based surveillance and elimination efforts demonstrated that malaria transmission in Botswana has become focal (Fig. 6). The country has made progress in confirming the diagnoses of presumptive cases, increasing from 63 to 90 % between 2012 [9] and 2014. High proportions (63.5 %) of the positive cases were reported through notification forms. However, the emergence of malaria outbreaks in a few districts (Kgalagadi North, Kgatleng, Lobatse, and Sowa) in 2013 and 2014 is an example of the fallout caused by the obstacles to successful malaria elimination. The country had some challenges such as importation and movements of infected individuals, asymptomatic infections and programmatic issues while integrating follow-up malaria interventions within the national framework.

Studies have shown the presence of asymptomatic infections in communities in low transmission areas can be important reservoirs hampering elimination [24, 25]. Reactive case detection is critical to identify asymptomatic malaria cases in communities and for monitoring progress towards elimination [26]. Reactive case detection has been used to reduce transmission in countries such as Iran [27], China [28] and Brazil [29]. Botswana needs to strengthen detection and treatment of asymptomatic infections to help eliminate, and prevent the reintroduction of malaria.

The country has embarked on an elimination campaign but the cases are still predominantly (94 %; 1079/1148) locally acquired compared to Swaziland (51 %) [30] and South Africa (>50 %) [31]. Transnational importation [69 cases (6 % of the notified cases)] from neighboring malaria endemic countries was one of the contributors of malaria movement while local movement [31 cases (2.7 % of all the notified cases)] between endemic and non-endemic districts of Gaborone, Kgatleng, Selebi Phikwe, South East and Kanye was relatively common. These imported cases are one of the challenges [32] in Botswana’s elimination efforts. Due to relatively high endemicity in neighboring (Fig. 2) countries [33, 34] cross border malaria control efforts with adjacent countries are essential to eliminate disease [35]. Districts in Namibia (Caprivi district bordering Okavango, Ngami and Chobe), South Africa (Limpopo) and Zimbabwe (Hwange) that share common borders with Botswana continued to report high incidence of malaria in 2011 and these rates increased in 2013 [36] (Fig. 2). The NMP, Botswana should strengthen Port Health Services to build capacity for testing immigrants from endemic areas and collaboration with regional countries through the regional initiative of SADC, the Elimination 8 (E8) which promotes cross border collaboration through joint planning, monitoring, information sharing and synchronization of implementation of activities across borders where practical and feasible.

Notification forms had information (patient information, travel history, diagnosis and treatment) that identified villages which could be potential transmission hot spots. However, health workers often failed to complete key sections such as demographic data, location of households with patients, travel history, housing structure and socio-economic status in the notification and follow-up forms [35]. Consequently, active surveillance and community follow up of asymptomatic cases were limited. Training, retraining, reporting systems using mobile phones, and incentives for case notification may help to overcome challenges.

The occurrence of malaria outbreaks in several districts demonstrates the critical need for properly trained epidemic control teams with transnational communication to deal with continued flare ups of disease as elimination is attempted.

For elimination purposes all suspected cases should be confirmed and treatment should be based on laboratory test results [37]. Botswana used Paracheck RDT that is specific to *P. falciparum* and has a sensitivity of 89 to 96 % and specificity of 50.4 to 80.0 %, thus the performance of this test could have led to problems estimating the true prevalence of malaria infections [38–41]. Consequently, other *Plasmodium* species would not be detected and low level parasitemias by *P. falciparum* might not be recognized during screening near positive cases - underestimating the real prevalence of infection. Botswana has not yet introduced gametocidal treatment in line with requirements for elimination due to difficulties in getting the drug registered by the Drug Regulatory Unit as no companies appear willing to complete the process. The efficacy of AL has declined elsewhere [42] and monitoring in Botswana needs to be improved to identify the development of parasite resistance. The country may need to incorporate an approach that uses PCR to boost quality control of diagnostics during patient follow-up after treatment with ACTs.

Sensitivity of vectors to chemicals is done annually at six sentinel sites for vector surveillance. The vectors are still highly susceptible to DDT.

There is also a need to identify treatment failures of malaria infection and differentiate it from new infections. This requires further efforts in follow up surveillance of diagnosed patients to the 28 day period and use of molecular tests during follow-up. Programmatic follow up is a substantial challenge, with health workers indicating inadequate human resources as the major limitation. In Botswana only 24.6 % patients were followed to evaluate parasite clearance. DHMT identified low IRS coverage (67 %) and LLINs ownership (72 %) that predisposed small farming areas in Okavango to that outbreak [12]. The Chobe, the outbreak affected mainly commercial farming areas and road construction workers where the residential structures for migrant workers were not suitable for IRS. Migrant workers also might serve as a potential source of infection. More LLINs and IRS in highly epidemic districts may reduce malaria epidemics [43].

The findings from case investigations in Botswana were similar to that reported in Swaziland, another country targeted for elimination where 67.4 % of the cases were investigated and screening around positive cases yielded only 2.02 % positives from 3671 contacts [30]. The NMP, Botswana should target newly identified, sporadic and oscillating hot spots for higher intervention coverage. The increase in cases in 2013 and 2014 malaria transmission season was also noted in neighbouring Zimbabwe [44]. The SADC Climate Service Centre predicted a normal to above normal rainfall for Botswana in 2013 and 2014. The increase in cases might result from the increase in rainfall [45].

At the district level, the programme lacks surveillance officers, resulting inadequate supervision of malaria activities, case investigation and follow-up in the districts. NMP, Botswana also needs to regularly update its malaria risk map [46–58] at household level. Low community uptake of interventions in some districts also impacted negatively on the implementation of malaria elimination activities by allowing transmission to become locally re-established. The national budget will need to be supplemented to support elimination activities by recruiting more staffs and especially with the need to improve quality control of reporting and follow-up of passively identified cases.

## Conclusion

Malaria transmission in Botswana was predominantly local. Identification of asymptomatic infections, socio-economic and ecological risk factors at individual and household level have become important and strengthened cross border collaboration are critical to achieve elimination. However, the country also needs improvement in program quality control and timely follow-up of the reports. Surveillance officers integrated into the system will strengthen malaria surveillance ensuring proper follow-up and documentation of elimination experiences. Therefore, continued sustained funding with adequate human resources will be crucial for successful malaria elimination from Botswana.
